# A Rare Case of Pedunculated, Prolapsed Juvenile Rectal Polyp in a Pediatric Patient

**DOI:** 10.7759/cureus.71997

**Published:** 2024-10-21

**Authors:** Danielle A Rowe, Kavita Bharrat, Kelon Scott, Bezawit Asore, William Middlesworth

**Affiliations:** 1 General Surgery, College of Medicine, American University of Antigua, Osbourn, ATG; 2 General Surgery, BronxCare Health System, Bronx, USA; 3 General Surgery, American University of the Caribbean School of Medicine, Cupecoy, SXM; 4 Surgery, American University of Antigua, St John’s, ATG; 5 Pediatric Surgery, BronxCare Health System, Bronx, USA

**Keywords:** blood per rectum, juvenile rectal polyp, pediatric polyp, prolapsed rectal mass, rectal polyp

## Abstract

The prolapse of a pedunculated juvenile rectal polyp is a rare event. It occurs when the polyp protrudes through the anus and appears as a fleshy mass at the anus, leading to an alarming scenario for both the parent and patient and typically prompting a visit to the emergency department (ED). We report a case of a five-year-old male patient who presented to the ED with a prolapsed rectal mass. A careful examination revealed it to be a pedunculated prolapsed polyp through the anus. The polyp was excised in the ED without complication, and the patient was discharged home with follow-up appointments with both pediatric surgery and gastroenterology for colonoscopy. The pathology report later confirmed the diagnosis of the juvenile polyp. We present this case to raise awareness about the various presentations of juvenile polyps and to familiarize clinicians with this diagnosis.

## Introduction

Juvenile polyps are benign hamartomatous polyps characterized by the cystic dilation of glandular structures within the lamina propria. They are among the most common types of colonic polyps in childhood, accounting for more than 90% of all colonic polyps in children [1]. They are typically limited to the rectosigmoid colon [2]. Affected children are typically asymptomatic; however, due to the generous vascular supply within the stroma, these polyps may present with rectal bleeding [3-7], which is the most common presenting symptom [6]. Juvenile polyps typically present as a single pedunculated lesion. The pedunculated structure of the polyp makes them susceptible to prolapse during defecation [4]. However, prolapse is still a rare occurrence in affected children [3,8].

Juvenile polyps may mimic other anorectal diseases such as hemorrhoids and rectal prolapse, and hence careful assessment is required to avoid misdiagnosis. The typical age of presentation is three to six years [5,6]; sporadic juvenile polyps are typically not seen before the age of two years and are considered rare [1]. Juvenile polyps are most commonly diagnosed during a colonoscopy. Polypectomy is required for its treatment and definitive diagnosis. While patients presenting with a solitary juvenile polyp are not at increased risk of developing cancer [6], those with multiple proximal gastrointestinal polyps need to undergo a screening colonoscopy [9]. We discuss a case of a five-year-old male patient who presented to the ED with a prolapsed mass through the rectum. A polypectomy was performed in the ED, which the patient tolerated well, and he was subsequently discharged home with follow-up appointments. Histologic evaluation confirmed the diagnosis of juvenile polyp. We present this case to raise awareness about this condition.

## Case presentation

**Figure 1:**
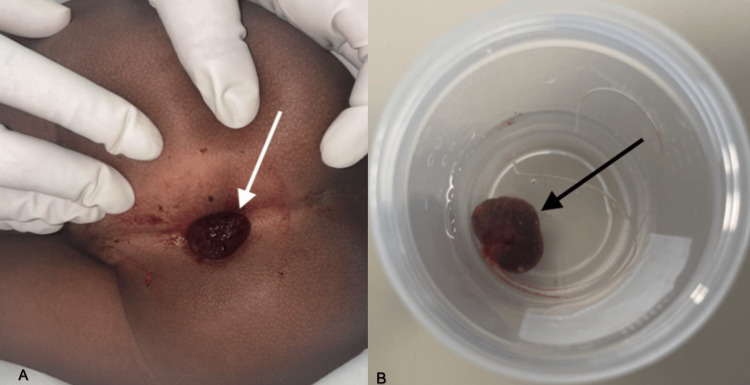
A: Gross image of buttock and rectum demonstrating violaceous prolapsed juvenile rectal polyp (white arrow). B: Excised prolapsed juvenile polyp measuring 2.3 x 1.6 x 0.8 cm (black arrow)

A five-year and seven-month-old male with no significant past medical history who was not on any medications presented to the pediatric ED after his father noted a purple mass at the patient's anus. The father stated that the patient had gone into the bathroom to have a bowel movement, and he had gone in to check on him as the patient had not emerged from the bathroom after an excessive amount of time. The father had noticed a mass protruding from the anus, which he had attempted to reduce but had been unable to. The patient had no history of rectal bleeding or abdominal pain, though he did report occasional constipation. Surgical consultation was sought by the pediatric ED team, for an evaluation of a prolapsed rectal mass. On physical examination, the patient appeared well, and in no acute distress. Vital signs were within normal limits - temperature: 98.5 °F, blood pressure: 107/56 mmHg, and pulse: 110 beats per minute. The abdomen was nondistended, soft on palpation, and nontender with normoactive bowel sounds. On rectal examination, a violaceous mass approximately 3 cm in diameter (Figure 1) was observed at the anus with a thin stalk extending from the posterior wall of the rectum. The mass was engorged.

**Figure 2:**
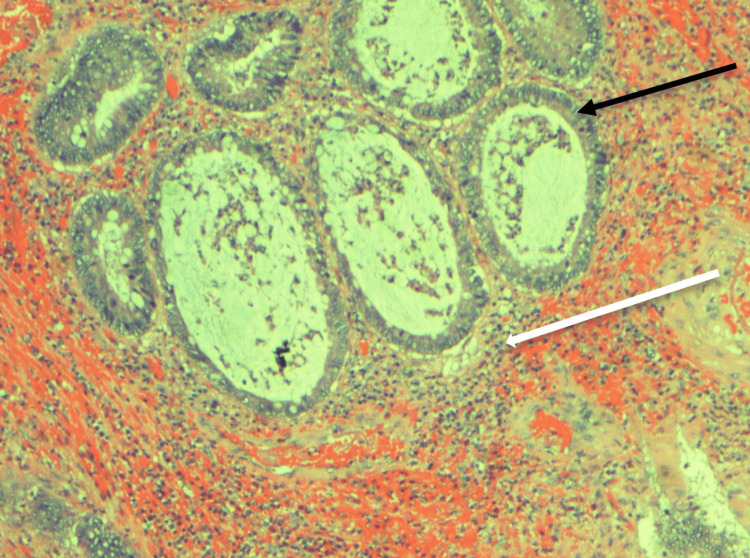
Hematoxylin and eosin stain (10x) revealed abundance of edematous lamina propria and inflammatory cell infiltrate (white arrow) and cystically dilated glands lined by cuboidal to columnar epithelium with reactive changes filled with mucin (black arrow)

Our initial assessment of the mass was a polyp versus an external hemorrhoid. Due to the presence of the stalk, juvenile polyp was at the top of the differential diagnosis list. Since the child was very cooperative and in no distress, we chose to perform a bedside excision of the polyp. The stalk of the polyp was doubly tied with 0 Vicryl and the stalk was cut between the ties using scissors. The polyp was then excised and sent for pathology. The patient tolerated the procedure well. He was observed for two hours with no subsequent bleeding noted. The patient was discharged home with follow-up appointments with pediatric surgery and gastroenterology for screening colonoscopy. Histologic analysis of the polyp revealed polypoid rectal mucosa with ulceration, and an abundance of edematous lamina propria with recent hemorrhage and inflammatory cell infiltrate (Figure 2). Cystically dilated glands lined by cuboidal to columnar epithelium, also with reactive changes and filled with mucin were observed, consistent with a juvenile polyp.

## Discussion

Risk factors for intestinal polyp formation in children include excessive meat consumption, male sex, age less than six years, and a positive family history [6]. Polyp formation from high meat consumption is thought to be due to carcinogenic substances such as heterocyclic amines and polycyclic aromatic hydrocarbons in meat that are released in the process of high-temperature cooking [8]. However, this theory is not well established and needs further investigation. Estrogen is thought to be protective against polyp formation [10], which may explain the higher incidence of polyps in males [11,12].

Pre-school children aged three to six years have a higher risk of juvenile polyp formation [13,14], thought to be due to the rapid intestinal growth at this age under the influence of various hormones [15]. Wang et al. [6] found a positive correlation between colorectal polyp formation in patients with juvenile polyps. In some cases, patients may present without any obvious risk factors. Our patient was male and less than six years old, which aligns with some of the known risk factors for juvenile polyp formation. However, he had no known family history of colonic polyps, and the patient's diet was noted to be well-balanced, including fruits and vegetables. Despite the absence of certain risk factors, his age and gender warranted further investigation into his condition.

While the exact pathogenesis of juvenile polyps is not fully understood, it is thought to be due to a local inflammatory response. The clinical presentation of the polyp depends on its size and location. Most polyps are found in the sigmoid or rectum (80-90% of cases) [2]. They typically measure 3 cm in diameter and are pedunculated in 90% of cases. The most common symptom is painless hematochezia [2,6]. Less commonly, patients may present with prolapse (as in our case) [3,8]. Patients may also experience abdominal pain and weakness [6]. Polyps that prolapse generally do so during defecation, and when wiping, patients notice blood and the protruding mass, which often prompts a visit to the ED.

Direct visualization and polypectomy are both diagnostic and therapeutic and are commonly achieved at endoscopy. Ultrasound may also be used for visualization in suspected cases [16]. Prolapsed polyps, when they can be safely excised, should always be sent for histologic evaluation to establish an accurate diagnosis. The definitive treatment is polypectomy, most commonly performed endoscopically. If endoscopic polypectomy is not safely achievable, surgical excision is indicated [7]. In our case, a long stalk and a very cooperative and calm child allowed for safe double ligation of the stalk and excision with subsequent discharge of the patient. Given the risk of juvenile polyposis syndrome [7], screening colonoscopy is recommended in these patients. This case highlights the importance of recognizing juvenile polyps even in the absence of typical risk factors, with prolapse being a rare but notable presentation. Early detection and intervention through polypectomy remain key to managing these cases.

## Conclusions

This report serves as a reminder to clinicians to consider juvenile polyps in the differential diagnosis when encountering pediatric patients with a prolapsed rectal mass. A careful physical exam is recommended to differentiate between rectal prolapse and a prolapsed mass. A follow-up colonoscopy is indicated to delineate the extent of the disease and to evaluate for juvenile polyposis syndrome. These patients should undergo follow-up colonoscopies to rule out juvenile polyposis syndrome.
